# Alport Syndrome: A Comprehensive Review

**DOI:** 10.7759/cureus.47129

**Published:** 2023-10-16

**Authors:** Avanti Adone, Ashish Anjankar

**Affiliations:** 1 Medicine, Jawaharlal Nehru Medical College, Datta Meghe Institute of Higher Education and Research, Wardha, IND; 2 Biochemistry, Jawaharlal Nehru Medical College, Datta Meghe Institute of Higher Education and Research, Wardha, IND

**Keywords:** anterior lenticonus, sensorineural (sn) hearing loss, x linked recessive, end stage renal disease (esrd), alport syndrome, type iv collagen

## Abstract

Alport syndrome is an genetic disorder that distresses the basement membrane of the kidneys and can also impact other organs, such as the cochlea of the inner ear and eyes. It is characterized by mutation causing abnormalities in the collagen within the basement membrane, which has a crucial role in the filtration process of the kidneys. These abnormalities lead to progressive kidney damage and often result in chronic kidney disease. In some cases of Alport syndrome, the abnormal collagen can also affect the cochlea in the inner ear, leading to sensorineural hearing loss. Additionally, changes in the ocular lens, named anterior lenticonus, can occur, causing vision problems. Alport syndrome can manifest differently among individuals, and its severity can vary. Some people may experience mild symptoms, while others may develop more severe kidney problems, including end-stage renal disease, which may need dialysis or kidney transplant. Treatment for Alport syndrome primarily focuses on managing its symptoms and complications. Regular monitoring of kidney function and blood pressure, along with medications to control hypertension, are crucial aspects of the management plan. In cases of severe kidney damage, kidney transplantation may be necessary. As with any medical condition, early detection and intervention can improve results and quality of life for persons with Alport syndrome. Therefore, if there is a family history of the disorder or any concerning symptoms, it is essential to seek medical attention promptly. Genetic testing can help confirm the diagnosis and identify affected family members, allowing for appropriate monitoring and management.

## Introduction and background

Alport syndrome commonly affects the glomerulus, eye, and cochlea. It manifests due to mutation in type IV collagen-(COL4A) (3,4,5). This mutation thereby affects the synthesis of *COL4A5*, *COL4A3*, and *COL4A4* genes thereby causing the clinical manifestations. This syndrome is named after Dr. Arthur Cecil Alport, who first described the condition in 1927. Type IV collagen is a structure similar to a sheet present in the basement membrane of glomeruli. It provides a substructure to the cells and forms barricades [1]. There exist three forms of this disease which include: (i) X-linked Alport syndrome, which is the most common and arises due to the mutation in *COL4A5* on chromosome X, (ii) Autosomal recessive Alport syndrome, and (iii) Autosomal dominant Alport syndrome [2].

Children with this disease have an increased risk for renal impairment, sensorineural hearing loss, and anterior lenticonus. Prompt diagnosis of this syndrome is imperative considering the phenotypic and genotypic intricacy of the disorder [3]. If left undiagnosed and untreated, mild microscopic haematuria can progress to proteinuria, followed by glomerulonephritis and renovascular hypertension, and ultimately result in end-stage renal disorder (ESRD). This occurs with time due to missing type IV collagen where the glomerular basement membrane becomes overly permeable and thin for erythrocytes to pass [4]. The results of renal biopsy show focal segmental glomerulosclerosis. Conductive deafness does not become evident at birth but rather develops gradually during adolescence, mostly in the age group of 12-18 years. The inner ear comprises the cochlea, accommodating the organ of Corti, which consists of hair cells attached to the basement membrane. Because of the mutation in the basement membrane, atypical collagen is produced, leading to the lack of ability to generate nerve signals and leading to sensorineural hearing loss [5]. Ocular findings are seldom present at birth but progress as patients age. The middle part of the lens starts to push into the anterior chamber as the lens capsule lacks integrity in the shape of the lens [6].

Type IV collagen exists as a heterotrimer. It contains alpha chains in the triple helix containing glycine subunits. A person carrying a mutant variant in the *COL4A5* gene experiences the influence on all its collagen IV A3, A4, and A5 heterotrimers. The presence of an auxiliary variant in either *COL4A3* or *COL4A4* might not aggravate the disease. In contrast, a female with a pathogenic variant in the *COL4A5* gene generally has approximately 50% of her heterotrimers affected. If she carries an additional variant in *COL4A3* or *COL4A4*, the percentage of affected heterotrimers increases to 75%. This increase in affected heterotrimers is associated with an increased risk of developing proteinuria [7]. *COL4A3* and *COL4A4* are located in a parallel orientation on chromosome number 2. Therefore, it is vital not only to provide symptomatic treatment to the affected patient but also to identify and treat all family members who are at risk of progressing towards ESRD and might need kidney transplantation. Initiating prophylactic treatment for the identified family members is crucial in managing the condition effectively. 

The X-linked dominant variant of Alport syndrome is the predominant presentation among affected individuals. Initially, this pattern of inheritance was hypothesized after a thorough scrutiny of extensive Alport family histories. Subsequently, it was confirmed by identifying the dominant Alport locus on the X chromosome's long arm (Xq22) using the analysis of polymorphic markers. This milestone was followed by a notable achievement, the discovery of a specific gene (*COL4A5*) responsible for a unique type IV collagen chain, also mapped to the Xq22 region. The breakthrough continued with the identification of mutations in *COL4A5* within three Alport families. Since those initial discoveries, a multitude of mutations in *COL4A5* have been unearthed, the majority of which are distinct and singular. It is worth noting, however, that *COL4A5* mutations have been identified in only around 50% of individuals with X-linked Alport syndrome. Furthermore, some of these patients have been screened for mutations in the regulatory region immediately preceding the 5' end of *COL4A5*, but these endeavours have yielded negative outcomes. Importantly, no mutations in *COL4A6* have been observed in Alport patients, except in cases involving diffuse leiomyomatosis, characterized by aberrant smooth muscle growth. This observation aligns with the absence of normal ca6(IV) appearance in the glomerular basement membrane (GBM). Currently, there are lingering questions regarding the possibility of another gene in the Xq22 region playing a role in Alport syndrome or the potential presence of hidden mutations within the *COL4A5* gene, possibly within introns. The exploration of deletions and other significant structural rearrangements is also under consideration as part of the investigation into this intricate genetic condition [8].

Immunohistochemistry analysis of renal biopsy tissues depicts usual and not abnormal α5 (IV) staining, making it tough to precisely diagnose Alport dominant Alport syndrome (ADAS) based only on clinicopathological findings. Consequently, genetic testing becomes essential for a final diagnosis. While ADAS has been measured as an exceptionally erratic condition, the number of reported cases has been growing in the past few years, indicating the possibility of undiagnosed patients. Traditionally, thin basement membrane nephropathy (TBMN) has been viewed as a benign condition in terms of clinical progression. Nevertheless, only a limited number of research studies have documented cases where some patients with TBMN exhibit proteinuria and renal dysfunction. Furthermore, recent investigations have unveiled that specific individuals carrying one mutated copy of the *COL4A3/COL4A4* genes might eventually develop a kidney condition known as focal segmental glomerular sclerosis (FSGS), expanding the range of kidney disorders associated with type IV collagen mutations. Consequently, there is still significant uncertainty regarding the diagnosis, prevalence, and clinical characteristics of patients harbouring heterozygous mutations in the *COL4A3*/*COL4A4* genes [9]. The disease follows a mode of inheritance where mothers with heterozygous genes act as carriers and possess a 50% probability of transmitting the disease in every new pregnancy. Male children who inherit the gene from their mothers have a definite chance of acquiring the disease and developing symptoms. However, daughters who inherit the gene may exhibit asymptomatic haematuria or complete kidney failure. If sons inherit the disease, they will transmit the variant to their daughters but not to their sons [10].

## Review

Methodology

A systematic search was undertaken within PubMed Central and Google Scholar in July 2023 using keywords like “Alport syndrome” and “Genetic background” and Medical Subject Headings (MeSH) as (((Alport Syndrome [Title/Abstract]) OR (GBM thinning [Title/Abstract])) OR ("anterior lenticonus" [MeSH Terms]) AND (("Pathogenesis" [Title/Abstract]) OR (QoL [Title/Abstract])) OR ("Genetic background" [MeSH Terms]). terms such as "Alport syndrome*" and "Alport synd*," in conjunction with MeSH terms. Moreover, key references were searched from bibliographies of the relevant studies. In addition to the diagnosis of Alport Syndrome, GBM thinning, and anterior lenticonus, the pathogenesis and quality of life (QoL) aspects were also investigated. To ensure thoroughness, articles related to the genetic background of these conditions were also included.

**Figure 1:**
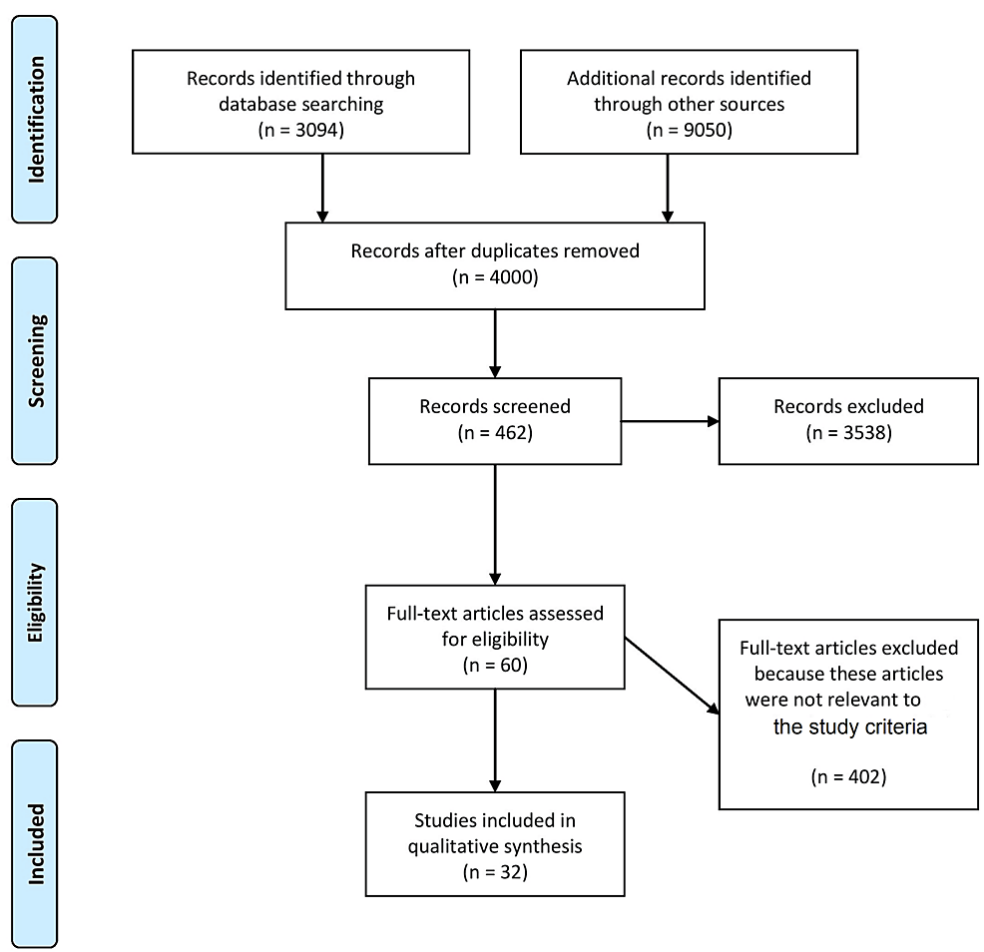
PRISMA flow diagram PRISMA: Preferred Reporting Items for Systematic Reviews and Meta-Analyses

A total of 462 relevant articles were screened. To focus on specific study types, we applied filters for clinical trials, meta-analyses, randomized control trials, and systematic reviews. Furthermore, we included studies with a target age of less than 40 years. One reviewer independently assessed the retrieved studies based on title and abstract, initially, followed by full-text evaluations. To ensure accuracy, a second reviewer reviewed approximately 20% of these studies to validate their inclusion. Any discrepancies were resolved through thorough discussions. Articles which satisfied the relevant study criteria were included while duplicate and non-relevant literature were excluded. The process is shown in the Preferred Reporting Items for Systematic Reviews and Meta-Analyses (PRISMA) flow diagram (Figure 1). 

Genetic background

Alport syndrome is the most prevalent hereditary kidney disease. It is more widespread than polycystic kidney disease, which is autosomal dominant. The autosomal dominant form of Alport syndrome affects 1:100 of the people. The X-linked form is more fatal and has an influence on one in 2000 of the population [11]. Patients who carry a single pathogenic variant in either *COL4A3* or *COL4A4* genes are diagnosed with either TBMN or ADAS.

After diagnosing Alport syndrome, it becomes crucial to differentiate between X-linked and autosomal recessive inheritance patterns due to their distinct implications, especially concerning the risk of renal failure within affected families. X-linked Alport syndrome is more prevalent, occurring about five times more frequently than the recessive form. In some cases, the mode of inheritance can be inferred from the family's pedigree. In X-linked inheritance, there might be instances where the disease seems to skip a generation, particularly when there is an affected female exhibiting hematuria without other associated features. In contrast, recessive inheritance patterns typically manifest within a single generation, affecting both males and females with equal frequency and severity. Furthermore, it's possible for the father of an affected individual to display hematuria. Suspicions of recessive inheritance are heightened when a young female presents with a combination of renal failure, hearing loss, and ocular abnormalities. Genetic testing is usually employed to confirm the mode of inheritance. Occasionally, the composition of collagen IV in the GBM is used for diagnostic purposes. However, this test is not widely accessible, and its results can be challenging to interpret, especially in females with X-linked Alport syndrome [12]. Heterozygotes with a pathogenic variant characteristically show signs such as haematuria and, every so often, proteinuria, hypertension, and kidney damage. However, they seldom exhibit hearing loss or ocular abnormalities, which are more characteristic of X-linked Alport syndrome. Furthermore, atypical features such as leiomyomatosis and vascular abnormalities can also be present in some cases [13].

Alport syndrome is chiefly triggered by two types of genetic variants. About 85% of cases are X-linked and result from pathogenic variants in the *COL4A5* gene. The remaining 15% of cases are autosomal recessive, this means that they arise from the mixture of two morbific variants in either the *COL4A3* or *COL4A4* genes on diverse chromosomes. The dangers associated with Alport syndrome are noteworthy. In men with a *COL4A5* variant, around 90% will suffer from kidney failure demanding replacement therapy by the age of 40. For women with a *COL4A5* variant, the risk of renal failure by the age of 60 is approximately 15-30% [14]. As for persons with recessive Alport syndrome, both men and women definitely experience kidney failure by the end of 20 years of age. It is of utmost importance that Alport syndrome must not be misdiagnosed with similar pathologies causing kidney dysfunction. The occurrence of pathogenic variants in these conditions is higher than expected by chance, indicating that these variants likely play a role in causing the diseases. Additionally, *COL4A3*/*COL4A5* variants are also present in cases of cystic kidney diseases, but only after autosomal dominant polycystic kidney disease has been ruled out [15]. Goodpasture syndrome also causes a similar set of symptoms as the Alport syndrome. It is an autoimmune disorder generating antibodies against GBM of the kidneys and alveoli of the lungs [16]. The clinical presentation, prevalence, and biopsy findings are mentioned in Table 1.

**Table 1:** Diagnostic features of Alport syndrome

Diagnostic category	Findings
Clinical Picture	Patient presents with hematuria, mild proteinuria, hypertension
Occurrence	1:50000 live births (rare disease)
Hereditary aspect	X linked dominant disorder having mutation in Alpha chain of type 4 collagen
Light microscopy	Glomeruli show segmental proliferation of mesangial cells with increased mesangial matrix and occasional segmental sclerosis. Lipid-laden foam cells in interstitium.
Electron microscopy	Characteristic basement membrane splitting or lamination in affected parts of glomeruli
Immunofluorescence	Fail to show deposits of immunoglobulins or complement components

Pathogenesis

The detection of Alport syndrome is vital due to the risk it poses to other members of the family and prophylaxis with angiotensin-converting enzyme (ACE) inhibitors, which postpone the commencement of renal impairment [17]. Collagen being the most plentiful protein in the body is accountable for membrane integrity and strength. X-linked Alport syndrome is caused due to various types of genetic mutations. These mutations include 40% missense mutations, nonsense mutations, and composite changes leading to 40% downstream nonsense changes and 10% splice site mutations. Mutations that are missense type frequently lead to the manufacture of misfolded proteins, which get stuck in the endoplasmic reticulum and are afterwards broken down through a cellular response known as the unfolded protein response. Nonsense type of mutations, like those hereditary collagen diseases, likely induce nonsense-mediated decomposition. This development consequences in the degradation of a major portion of the corresponding mRNA, preventing the synthesis of abnormal proteins [18].

Alport syndrome is usually not diagnosed by many nephrologists and is often missed. It was the first genetically acquired kidney disease for which hereditary bases were discovered. It is also known as thin basement membrane disease. Haematuria was found in 92% of the known cases. Of the patients, 65% had proteinuria and needed a kidney transplant. The prognosis was better in patients who received kidney transplants [19]. Some of the extrarenal symptoms in Alport syndrome are conductive deafness or sensorineural hearing impairment, retinal stippling, anterior lenticonus, oesophageal leiomyomatosis, and macrothrombocytopenia [20]. Alport syndrome may also result in perimacular retinal flecks. Ocular appearances of Alport can be a source of retinopathies. A study indicates 93.7% (n=30) of the study participants had a family history of renal disease. All the patients in the study had some form of renal condition. Specifically, 56.3% (18 patients) had chronic renal failure, 12.5% (four patients) had renal deficits, and 31.3% (10 patients) presented with haematuria. Sensorineural deafness was found in 62.5% (20 patients) of the cases. Additionally, 40.6% (13 patients) exhibited ocular deformities, with 15.7% (five patients) manifesting typical ocular changes, including three having anterior lenticonus and two having macular flecks [21].

X-linked Alport syndrome happens due to mutations in the *COL4A5* gene. Males having a hemizygous genotype show a 100% likeliness of progressing towards ESRD, with the rate of evolution and clinical representation of extrarenal appearances being predisposed by their specific *COL4A5* genotype. On the other hand, females with a heterozygous genotype have an approximate 25% lifetime risk of developing ESRD. Numerous elements contribute to the likelihood of disease progression in females who carry one copy of the X-linked Alport syndrome gene. These factors encompass instances of noticeable blood in the urine during childhood, sensorineural deafness, protein leakage in the urine, and substantial thickening and layering of the GBM [22]. In individuals with Alport syndrome, particularly males, the lens capsule is weakened and lacks the necessary structural strength to retain the typical shape of the lens. As a consequence, over time, the central part of the lens bulges forward into the anterior chamber of the eye, a condition known as anterior lenticonus. This occurrence is gradual and takes place over several years [23]. Alport syndrome disrupts the usual formation of the collagen IV α345 network in the basement membranes of the cochleae, resulting in an abnormality. However, the precise mechanism by which this irregularity causes hearing loss in affected individuals is not yet fully understood [24].

Diagnosis

**Figure 2:**
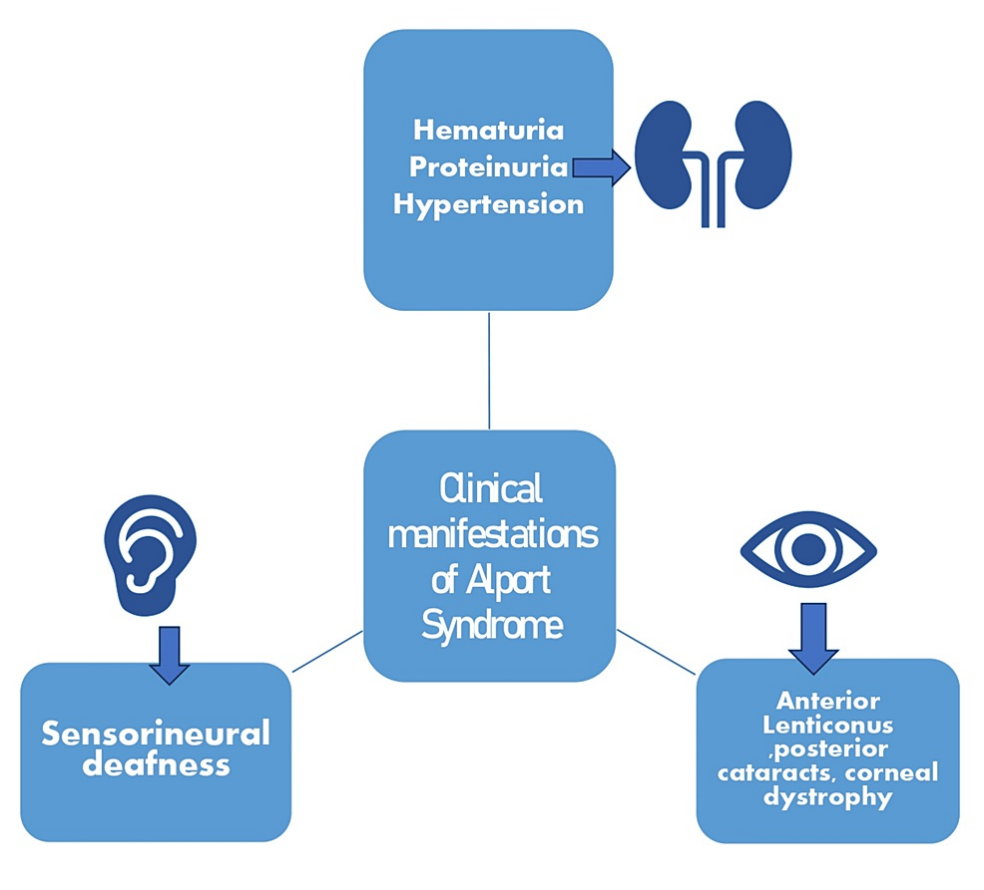
Clinical manifestations of Alport Syndrome Image Credit: Author

Thorough history taking is important as there may be first-degree family history of hematuria without leading to kidney failure, ocular signs or hearing issues, and renal ultrastructure changes. Clinical examination is always done when the patient presents with signs and symptoms of hearing loss, kidney problems, and problems with vision. A urine sample will be analyzed to look for the presence of protein, blood, bile salt, bile pigment, and glucose [25]. Blood tests will be performed to evaluate kidney function and identify any electrolyte imbalances. A hearing test will be administered to assess hearing function. Genetic testing is essential to confirm specific gene mutations linked to Alport syndrome. This is especially important for identifying carriers and offering genetic counselling to family members. The diagnosis of Alport syndrome involves a multi-faceted approach, which includes clinical assessment, the possibility of renal or skin biopsy, also the gold standard of genetic screening. Genetic screening is particularly valuable as it can pinpoint specific genetic mutations linked to the condition [26]. Renal and skin biopsy should be performed to confirm the diagnosis and assess renal damage. To avoid the use of invasive procedures such as kidney and skin biopsy in young children, genetic analysis is done in the initial stages of diagnosis [27]. Diagnosing Alport syndrome in children can lead to psychological challenges for both the child and their family. Unlike asymptomatic adults from known Alport syndrome families, who may undergo diagnosis for kidney donation or genetic counselling without hesitation, it is generally not recommended for asymptomatic children. However, in cases where children present with persistent microscopic haematuria often accompanied by episodes of visible blood in the urine (gross hematuria), there may be significant anxiety for both patients and their families, leading to a strong desire for a definitive diagnosis [28]. The analysis of skin biopsy using immunofluorescence or immunoperoxidase methods can provide valuable insights into certain patients. This sampling approach is relatively straightforward and minimally invasive. It relies on specific observations: in the absence of skin conditions, the epidermal basement membrane (EBM) typically contains α5 but lacks α3 or α4. Consequently, in cases of the autosomal recessive form of the condition, α5 is found in the EBM, just as it is in normal individuals. The assessment and interpretation of results from renal biopsies, which encompass light and electron microscopy, as well as immunofluorescent or immunoperoxidase techniques, have been utilized. The focus is on their significance in establishing a diagnosis and distinguishing between the X-linked and autosomal recessive forms of the condition [26]. Figure 2 helps to understand the clinical presentation of the patient.

Treatment and management

The management of Alport syndrome includes treating symptoms and offering counselling to both the patient and their family. It is vital to maintain ideal blood pressure, which is typically achieved through the use of medications like ACE inhibitors or angiotensin 1 or 2 receptor blockers (ARBs). ACE inhibitor therapy has been proven effective in patients with proteinuria and well-preserved kidney function has been shown to suspend progression to renal failure [29]. Studies in animals with Alport syndrome have also confirmed that treatment that postpone the commencement of proteinuria or decrease its occurrence can extend kidney survival and prevent ESRD. Therefore, experts mention using medications that decrease proteinuria to manage the disease effectively. Regular monitoring of kidney function through blood and urine tests is crucial for timely intervention in case of damaging kidney function. While there are no officially approved treatments for Alport syndrome based on the information available, there are described therapeutic strategies that hold promise for retarding disease progression and enhancing life expectancy. Possible therapeutic options include paricalcitol, sodium-glucose co-transporter-2 inhibitors, bardoxolone methyl, anti-microRNA-21 oligonucleotides, recombinant human pentraxin-2, inhibitors targeting lysyl oxidase-like-2, hydroxypropyl-b-cyclodextrin, sodium 4-phenylbutyrate, and the investigation of stem cell therapy.

Challenges in Management

Alport syndrome frequently goes unnoticed or misdiagnosed due to its rarity and the variability in its clinical presentations. A complete understanding of the disease's pathophysiology remains elusive, and there are gaps in our comprehension of its genetic underpinnings. Consequently, further research is imperative to establish more efficacious therapeutic protocols. Individuals with Alport syndrome should seek guidance from healthcare professionals well-versed in the latest advancements within the field to access the most current information and treatment options [30]. Additionally, a low-protein diet may be recommended to reduce pressure on the kidneys and limit protein excretion in the urine. For patients with hearing loss, appropriate hearing aids or assistive devices can enhance communication and overall quality of life. Frequent eye check-ups are vital to address potential eye abnormalities related to Alport syndrome and prevent complications [31]. Genetic counselling is a vital component for patients and their families, helping them comprehend the inheritance pattern and the risk of passing the condition to future generations. Supportive measures like pain management, physical therapy, and psychological support play an imperative role in helping patients cope with the challenges of living with a chronic condition like Alport syndrome. In advanced stages of kidney failure, when kidney function is severely impaired, renal replacement therapy options such as dialysis or kidney transplantation may be considered as a treatment approach [32].

## Conclusions

Alport syndrome is a genetic disorder that primarily affects the kidneys and can lead to hearing and eye problems as well. This syndrome is caused by mutations in *COL4A* (3,4,5) genes responsible for producing collagen IV, a critical component of the body's connective tissues. Collagen is an essential protein found in various tissues, including the kidneys, inner ear, and eyes. Mutations in the genes encoding type IV collagen result in the abnormal structure and function of these tissues. As a result, the kidneys' ability to filter waste products and excess fluids from the blood is impaired due to thinning of GBM. Alport syndrome is usually inherited in an X-linked pattern, which means the faulty gene is located on the X chromosome. This pattern primarily affects males, who have one X chromosome and one Y chromosome. Females, with two X chromosomes, can be carriers of the mutated gene without experiencing severe symptoms themselves. However, in some rare cases, Alport syndrome can also be inherited in an autosomal recessive or autosomal dominant manner. There is currently no cure for Alport syndrome, but treatments focus on managing symptoms, slowing the progression of kidney disease, and addressing hearing and vision problems. This may include medications to control blood pressure and proteinuria, hearing aids or cochlear implants for hearing loss, and regular monitoring by healthcare professionals to assess kidney function and overall health. In some severe cases, kidney transplantation may be necessary for ESRD. Early detection and comprehensive management are key to optimizing outcomes and improving the long-term prognosis for individuals with Alport syndrome.
